# Learning About the History of Landscape Use for the Future: Consequences for Ecological and Social Systems in Swedish Bergslagen

**DOI:** 10.1007/s13280-012-0369-z

**Published:** 2013-03-10

**Authors:** Per Angelstam, Kjell Andersson, Maths Isacson, Dmitri V. Gavrilov, Robert Axelsson, Mattias Bäckström, Erik Degerman, Marine Elbakidze, Elena Yu. Kazakova-Apkarimova, Lotta Sartz, Stefan Sädbom, Johan Törnblom

**Affiliations:** 1 Faculty of Forest Sciences, School for Forest Management, Swedish University of Agricultural Sciences, PO Box 43, 730 91 Skinnskatteberg, Sweden; 2 Department of Economic History, Uppsala University, Box 513, 751 20 Uppsala, Sweden; 3 Institute of History and Archaeology, Ural Branch of Russian Academy of Sciences, Yekaterinburg, Russian Federation; 4 Faculty of Forest Sciences, School for Forest Management, Swedish University of Agricultural Sciences, PO Box 43, 739 21 Skinnskatteberg, Sweden; 5 Bergskraft Bergslagen, Harald Olsgatan 1, 714 31 Kopparberg, Sweden; 6 Man-Technology-Environment, Örebro University, 701 82 Örebro, Sweden; 7 Department of Aquatic Resources, Institute of Freshwater Research, Swedish University of Agricultural Sciences (SLU), Pappersbruksallén 22, 702 15 Örebro, Sweden

**Keywords:** Environmental history, Forest, Water, Mining, Regional studies, Sustainable development

## Abstract

Barriers and bridges to implement policies about sustainable development and sustainability commonly depend on the past development of social–ecological systems. Production of metals required integration of use of ore, streams for energy, and wood for bioenergy and construction, as well as of multiple societal actors. Focusing on the Swedish Bergslagen region as a case study we (1) describe the phases of natural resource use triggered by metallurgy, (2) the location and spatial extent of 22 definitions of Bergslagen divided into four zones as a proxy of cumulative pressure on landscapes, and (3) analyze the consequences for natural capital and society. We found clear gradients in industrial activity, stream alteration, and amount of natural forest from the core to the periphery of Bergslagen. Additionally, the legacy of top-down governance is linked to today’s poorly diversified business sector and thus municipal vulnerability. Comparing the Bergslagen case study with other similar regions in Russia and Germany, we discuss the usefulness of multiple case studies.

**Electronic supplementary material:**

The online version of this article (doi:10.1007/s13280-012-0369-z) contains supplementary material, which is available to authorized users.

## Introduction

Man’s impact on landscapes creates path dependence effects of importance both for sustainable development as a societal governance process, and for sustainability. Already Marsh (1864) stressed the need to study the transformation of the interaction of humans and the natural environment, as a tool for extracting historical lessons to help address today’s environmental problems (see Lowenthal 2000). The interest in understanding the history of landscapes as social–ecological systems in the context of sustainability and sustainable development has appeared in many disciplines (Lee 1993; Balée 1998; Franklin and Blyton 2011) within the “two cultures” of human and natural sciences sensu Snow (1993).

Environmental history has been addressed by both cultures, however, with limited integration between them (Merchant 2005). Worster (2005) argued for the need to focus on three aspects of landscapes. The first is the natural environments of the past. How did ecosystems develop in terms of their composition, structure, and function? The second is the human modes of production including technologies and ways of organizing production. How did the material culture of a society develop? The third is about perception, ideology, and value. What did people think about the non-human world? Altogether this means that there is a need to understand the role of the human being in the ecosystem. These three dimensions are very similar to the landscape concepts’ biophysical, anthropogenic and perceived dimensions (Grodzynskyi 2005; Angelstam et al. 2013a).

Another relevant discipline is economic history. This is a broad human science discipline with its main emphasis on empirical studies (Magnusson 2000; Olivier 2007). In most countries it is a sub-discipline within economics or history. In Sweden it is a discipline on its own, with close links to business economics, political history, and human geography. How resources are created, used, and allocated is studied (Magnusson and Serrano Pascual 2007; Magnusson 2011). The research often involves comparisons of companies, social groups, genders, regions, and countries (e.g., Ågren 1998). Historical transitions and their causes are important, but what survives or changes more slowly is also of interest. Of special interest are also society’s formal and informal rules—its institutions—and their role to create and maintain stability or contribute to a renewal of enterprises, patterns of subsistence, and living conditions (North 1990; Schön 2010). Agrarian (Myrdal and Morell 2011), forest (Agnoletti and Anderson 2000), and industrial history (Isacson 2007; Schön 2010) are examples of established sub-fields within economic history.

The biophysical landscape that provides natural resources has not been a primarily field of interest for economic historians. Indirectly, however, through the knowledge of business, technology, and methods of production, research gives an opportunity to deepen the understanding of the state and appearance of landscapes, including how they have been formed and transformed. Historical ecology is an effort to foster collaboration among social science disciplines (anthropology and geography) and several hybrid fields (environmental history, environmental sociology, human ecology, landscape ecology) (Balée 1998; Egan and Howell 2001). The need for collaboration among disciplines with a historic perspective has been manifested in historical geography since more than 200 years (Butlin 1993). Archeological and vegetation history studies help us to extend the temporal dimension. Researchers from vegetation sciences may through pollen analysis also search for traces of heavy metals and poisons from old furnaces, ironworks, sawmills and pulp mills in mashes, bog lands, and lakes (Agnoletti and Anderson 2000). Biologists and forest scientists can study changes of the contemporary flora and fauna and the transformation of naturally dynamic landscapes to production units. Researchers from different disciplines could together provide a deeper understanding of the historic transformation of landscapes’ ecological and social systems, and give a better opportunity to restore and create sustainable landscapes.

The importance of understanding the history of resource use, economy, and social issues for contemporary landscapes can be seen in the production of metals. Historically, this required integration of use of ore, water, and wood for bioenergy and construction, as well as of societal actors (Wagner and Wellmer 2009). The Swedish informal region Bergslagen is a good example of this integrated use of natural resources and society (Geijerstam and Nisser 2011). However, legacies of the past are important to contemporary economic, ecological, social, and cultural issues. Since about 2005, global demand for metals has increased as several Asian countries develop from rural to industrial societies (Richards 2009).

The increase in metal and mineral prices has stimulated renewed interest in the mineral resources in the Bergslagen region. Old mines are being re-opened, prospecting is advancing and several concessions for mining are under application or have been granted. Another contemporary issue in Bergslagen is the need for biodiversity restoration in terrestrial and aquatic systems, which is required by current EU and national policies (Angelstam et al. 2011b). Loss of industry jobs have triggered interest in rural development and the role of entrepreneurship for development (Geijerstam and Nisser 2011; Tillväxtverket 2011). Finally, the role of culture is stressed both regarding cultural heritage as a base for tourism destination development, and as a factor affecting social capital (Axelsson et al. 2013a). These issues have promoted an increasing interest in landscape history. This involves the geographies of landscapes as spaces and places at multiple spatial scales over time (Grodzynskyi 2005), power and control affecting governing and governance (Balée 1998), as well as benchmarks for the ecological restoration of natural biophysical environments (Angelstam et al. 1997; Wohl 2004, 2005; Angelstam et al. 2004a, 2011a, b, Angelstam et al. 2013b).

This study focuses on the Swedish Bergslagen region (e.g., Nelson 1913), and views the historical use of ore, forest, and water, as well as societal development as a case study of landscape history. We analyze (1) the historical phases of Bergslagen’s development, which was triggered by metallurgy and associated use of natural resources, (2) the geographical location and spatial extent of Bergslagen as a proxy of cumulative pressure on landscapes, and (3) the consequences of historical landscape use for natural capital and society. Finally, illustrated by a comparison of Bergslagen with Harz in Germany and the Ural Mountains in Russia, we highlight how one can expand the single case study approach by comparisons among multiple case studies to learn from the history of landscapes for integrated management and adaptive governance of natural resources.

## Materials and Methods

### Bergslagen as a Case Study of Landscape History

Bergslagen is an informal region in south-central Sweden (ca. 59–61°N latitude, 13–15°E longitude) with a history of industrial landscape use that began with production of metals more than 2000 years ago (Nelson 1913; Berger et al. 2006; Isacson et al. 2009). The name Bergslagen comes from the words mine (berg in Swedish) and law or team (lag in Swedish). Landscapes have had different roles over time in Bergslagen, and they form a gradient between rural remote regions and more developed urban areas. It ranges from Mälardalen valley’s agricultural landscapes and urban centers to the forested upland areas in the north (Nelson 1913). While the former began to be transformed by agricultural development several thousand years ago, areas immediately to the north were permanently colonized by agriculture only during the late 1500s when immigrating Finns settled to practice slash and burn farming (Nordmann 1888; Montelius 1953, 1962; Emanuelsson and Segerström 2002). Forests also provided grazing for cattle, fuel, and materials for buildings, fences, tools, and household items. Thus, there has been a long history of competing demands of goods, functions, and values in Bergslagen (Herou 2000; Andersson et al. 2012a, b).

### Methods

Focusing on the use of ore, forests, and water, we first reviewed literature about the historic phases of the economic and social development in the Bergslagen region. To map the location of Bergslagen, 22 different maps defining the spatial location and extent of Bergslagen (Electronic Supplementary Material, Table S1) were produced using ArcGis. Printed maps were scanned and rectified, and the border of Bergslagen then digitized. The locations of the different definitions were then delimited with parishes as the minimum mapping unit. This was made to minimize errors linked to the variable quality of maps. Then the number of spatial definitions of Bergslagen per parish was counted and presented as a map showing the gradient in four zones from the core of Bergslagen (with many overlapping spatial definitions), to the periphery of Bergslagen (with few spatial definitions). The number of definitions was thus used as a proxy for the gradient from stronger impact of metallurgy on social–ecological systems in the center of the region to weaker in the periphery. To understand some consequences for landscapes in the core and periphery of Bergslagen, we compiled proxy data linked to metallurgy, forests, and water, as well as for societal development. Regarding mining we compiled data from the Swedish National Heritage Board about the location of historical mines and furnaces (digitally available at Fornsök^1^). Regarding forests we mapped the human footprint on natural forests as the proportion of remaining unmanaged forests using formally protected areas as a proxy (e.g., Angelstam et al. 2004b; data from Swedish Environmental Protection Agency^2^). Concerning aquatic ecosystems we mapped the density of dams per 100 km^2^ of catchment area using the Swedish Water Archive’s data base hosted by Swedish Meteorological and Hydrological Institute^3^ (SMHI). Finally, the societal dimension was illustrated by the vulnerability of municipalities in terms of a high dependency of jobs created by few firms, and rankings of municipalities with respect to different aspects of business climate (Tillväxtverket 2011) in different parts of the Bergslagen region. Because all data represent total counts statistical analyses were not performed.

## Results

### Phases of Landscape History

Mines, forests, and streams in Bergslagen formed the natural capital base for economic development for all of Sweden for a long time (Heckscher 1935–1949), albeit in different phases. Small-scale production of iron began more than 2000 years ago in this region (Geijerstam and Nisser 2011). Mining for copper started in the eighth century, and in 1347 it was described how charcoal production at the mine in Falun, the main mining city in the region, was managed (Söderberg 1932; Rydberg 1982). Industrial iron mining commenced during the early Medieval period (Bindler et al. 2011).

Until the beginning of the seventeenth century most of the mining in Bergslagen was performed on a seasonal basis by peasant miners (Bergsmän in Swedish). They had special privileges that included tax reductions and sometimes exemption from military service. The entire family was engaged with farming during the cultivation season and with mining and pig iron production the rest of the year. Thus, Swedish iron production was co-operatively organized (Montelius 1959; Geijerstam and Nisser 2011). The special privileges for “Bergsmän” gradually disappeared between 1810 and 1860, and mining became a small to medium size company business.

A vital contribution to the economy came from wood production and transportation to and from the abundant mines and blast furnaces in the area (Arpi 1951; Eriksson 1955). Before gunpowder was introduced in mines in the eighteenth century, immense quantities of firewood were necessary to crack the rocks and set free the ore. Wood was also used in mines as props, stairs, and hauling installations. In the next phases of production of iron and copper, large quantities of charcoal were consumed when the ore was roasted, melted in the blast furnaces and when pig iron was refined and hammered out to bar iron in the forges.

**Fig. 1:**
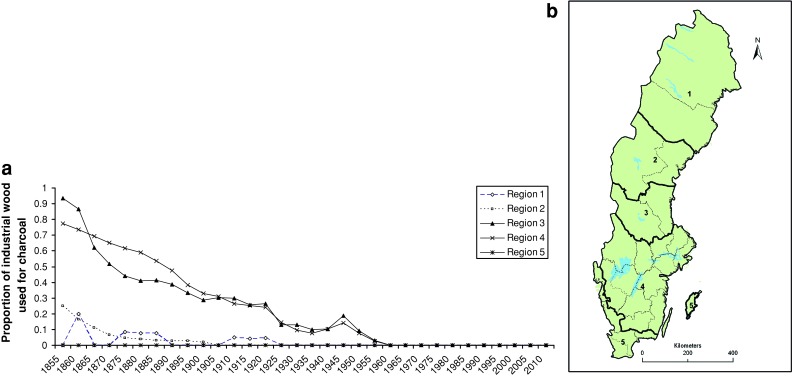
Regional variation over time of the proportion of all industrial wood (sawlogs, pulpwood, veneer and wallboard, charcoal wood and roundwood export) (**a**), which was used for charcoal production (data from Arpi 1959, 202 ff., and Skogsstyrelsen’s annual yearbooks). Bergslagen is located at both sides of the border between regions 3 and 4 (**b**), see Fig. 2

From the beginning of the seventeenth century, when the demand of iron from abroad increased, iron masters (Brukspatron in Swedish, i.e., merchants and noblemen as owners) paralleled the “Bergsmän” cooperative organizations. Subsequently the former gradually took over the bar iron production, which was the most profitable part. Large ironworks were established in Bergslagen. Swedish ironmasters who wanted to increase their production of bar iron were obliged to buy privileged quantities from other ironmasters, or build new ironworks outside the core of Bergslagen (Furuskog 1924; Eriksson 1955; Hildebrand 1992). The spatial expansion of the iron production from the end of the seventeenth century took place in the periphery of Bergslagen, and in other parts of Sweden. Iron ore and limestone were transported on land and water many hundreds of kilometers from mines in Bergslagen to the new ironworks. By contrast the charcoal transport was too expensive, and the charcoal too brittle to be transported more than about 30 km on sledges without being broken into too small of pieces (Arpi 1951; Geijerstam and Nisser 2011). From the 1860s, charcoal could be transported by railway from distant forests where the production was done in coal ovens (Arpi 1951; Isacson 1998a, b). Around 1900 forests were still used for charcoal production, but gradually to a much lesser degree as forestry became more profitable than iron production (Eriksson 1955). Thus, forests became important for sawmilling and later for the pulp and paper industry. The charcoal consumption dwindled rapidly, and this transition process finished in the late 1950s (Fig. 1).

Up to the mid-twentieth century the mining region of Bergslagen was a working landscape (Ågren 1998). The farming families were seasonally occupied in the mines or in the forests to produce charcoal, used streams to produce energy, and worked in the fields to produce food. This changed completely in the beginning of the 1950s, connected to the effective rationalization of forestry (Ager 1992). As a consequence families left their small farms and moved to industrial cities. The past working landscape of Bergslagen changed to a leisure landscape where people returned for vacations.

This long landscape history of industrial use of natural resources, that once provided many local jobs, resulted in relatively low levels of entrepreneurship and education among local people compared to regions with more diversified livelihoods (Bergdahl et al. 1997). Currently, natural and cultural landscape values are emerging as providers of post-modern products in terms of tourism and amenity migration (Vail and Hultkrantz 2000). Natural resources still, however, continue to be a base for commodity production based on wood, metal, and water in Bergslagen, but immaterial values are becoming increasingly important for rural development.

### Predicting Footprints from Bergslagen’s Core to Its Periphery

**Fig. 2:**
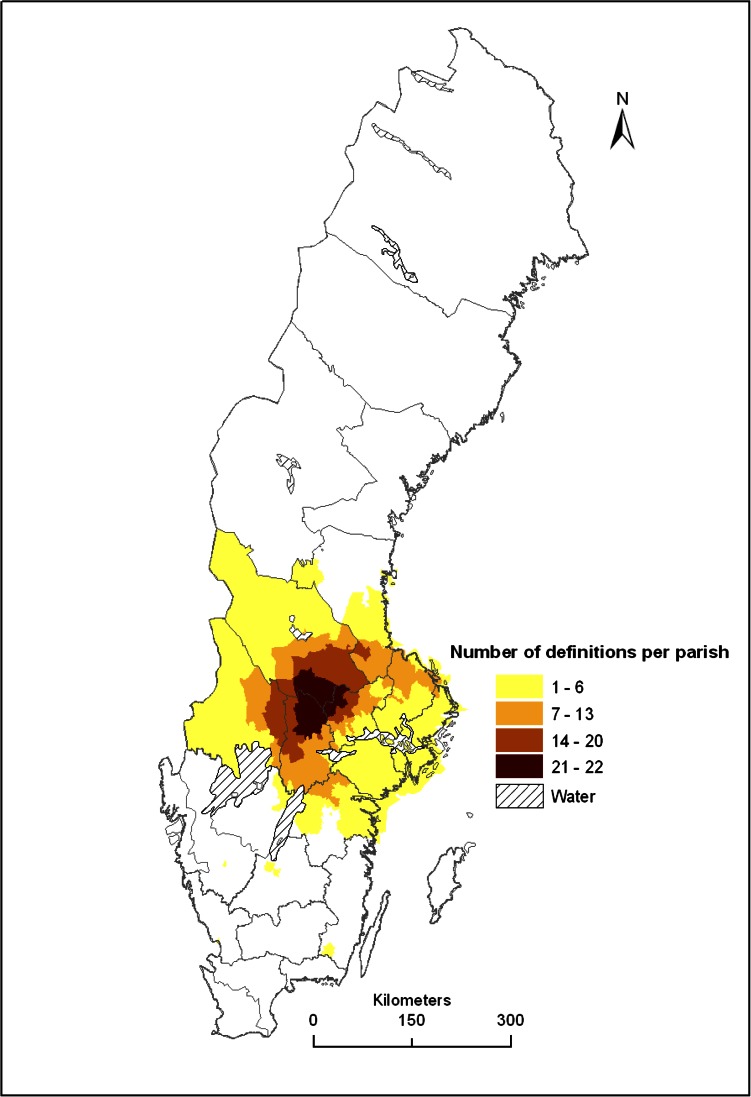
Map showing the location of the Bergslagen region according to different number of combinations (1–6; 7–13; 14–20; 21–22) of totally 22 different definitions of Bergslagen in Sweden (see Electronic Supplementary Material, Table S1)

Based on 22 polygons representing different definitions of the location and extent of Bergslagen (Electronic Supplementary Material, Table S1), 704 parishes with different number of spatial definitions were identified. These data were used to define four spatial zones from the core to the periphery of Bergslagen (see Fig. 2). The core of the Bergslagen region was determined as the parishes that coincided with 21–22 definitions covering a total of 553 000 ha, and the union of all 22 definitions from the core to the periphery in four zones covered 11 123 000 ha. We predicted that these four zones could be used to rank different parts of Bergslagen from more to less affected by landscape history.

### Consequences of Landscape History on Social–Ecological Systems

#### Mining and Metallurgy

**Fig. 3:**
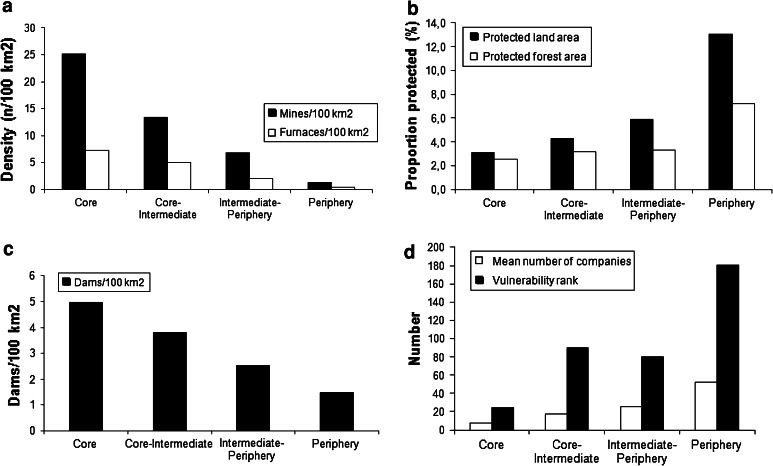
**a** Mines and furnaces in different zones from the core to the periphery of Bergslagen (see Fig. 2); data from the Swedish National Heritage Board. **b** Proportion of formally protected areas (national park, nature reserves, and biotope protection) in different zones from the core to the periphery of Bergslagen (see Fig. 2); data from the Swedish Environmental Protection Agency’s data base. **c** Number of dams per 100 km^2^ in different zones from the core to the periphery of Bergslagen (see Fig. 1); data from SMHI, the Swedish Water Archive data base on dams. **d** Average number of companies producing goods or services required to reach 50 % of the total income of individuals (*white bars*) and vulnerability rank (*black bars*) of municipalities located from the core to the periphery of Bergslagen (see Fig. 2); data from Tillväxtverket (2011)

The oldest mine in Sweden is the copper mine of Falun in north Bergslagen. Here mining began in 480–670 AD or earlier (Eriksson and Qvarfort 1996). Most Swedish ore deposits were discovered in the sixteenth century. Iron then became the main base for mining until the decline phase during the second half of the twentieth century (Geijerstam and Nisser 2011). The density of mines per unit area then decreased rapidly from the core to the periphery of Bergslagen (Fig. 3a). For furnaces the pattern was the same but the decrease was slower. Even though today all but three are closed, the long history of mining led to a legacy of polluted water, soils, and remaining sludge deposits (Bindler et al. 2009; Sartz 2010). The environmental impacts from mining include the release of acidic mine drainage, which leads to acidification of soil and water, and metal contamination of water, soil, and sediments (Lemly 1994). In Bergslagen several old mining sites are considered to pose great risk to the environment and public health (Allard et al. 2008). Surface water is greatly affected, mostly by copper, but at some sites also a risk to human health has been recognized (primarily because of arsenic pollution) (Sandén et al. 1987).

#### Forest

Initially wood harvesting was done using dimension fellings, meaning that big trees were felled and the rest were left behind. This produced forests with a low standing wood volume and poor growth rate, which forced the forest-dependent and nationally important mining and iron industry to recognize the need for efficient sustained yield wood production already in the late eighteenth century (Wieslander 1936; Almquist et al. 1980; Brynte 2002). A short rotation time was used due to the focus on the most important products—wood fuel for mining before the appearance of modern explosives, and charcoal for iron production. Thus, there were few stands older than 60 years, an optimal rotation time for charcoal production (Ek 1995). When the mining industry ceased to be the major purchaser of wood, companies’ focus gradually evolved to producing timber and pulpwood (Eriksson 1955; Arpi 1959). The age distribution was therefore shifted toward tree dimensions that satisfied the sawmilling industry, and resulted in older forests than during the charcoal period (Angelstam et al. 2011a). Today the principle of forest management is to maintain a maximum sustained wood yield by using clear-felling methods (Axelsson et al. 2007; Axelsson and Angelstam 2011; Elbakidze et al. 2013), and the main end-user is the export-oriented forest industry. This long history of forest use is linked to a clear spatial gradient of increasing amounts of near-natural forest patches from the center to the periphery of Bergslagen (Fig. 3b).

#### Water

The effects of mining on water include direct toxic effects of metals, acidification from sulphidic ores, and habitat degradation such as dams that were built and streams which were cleared for timber floating (Hoover and Hoover 1912; Lemly 1994; Jakobsson 1996). The acid rock drainage from sulfide ores, further enhanced by oxidation processes for the extraction of the metals, lowered the pH of surface and ground waters. Impacted streams were probably void of fish during the active period, and even afterwards (cf. Farag et al. 2003). Additionally, abandoned mines may still cause pollution (Younger 2001). Falu copper mine, in the midst of Bergslagen, was the largest producer of copper in Sweden and the environmental effects on the surroundings have been thoroughly mapped (Ek et al. 2001). Nearby rivers and lakes were void of fish for hundreds of years (Lindeström 2002). This was probably not only due to toxic levels of heavy metals, but also acidified waters due to acid mine drainage (Ek and Renberg 2001). Mills for producing power for dewatering arrangements, rod-engines, sawing, and bellows, were built wherever possible. There was a steep gradient in the density of dams from the center to the periphery of Bergslagen (Fig. 3c). Thus, the amount of migration obstacles in Bergslagen’s rivers and streams caused by remnants of old dams or dams still in use is considerable.

#### Municipal Vulnerability

Forming the smallest unit for democratic governance in Sweden, individual municipalities’ vulnerability has been a recurring issue at least since the 1970s and its industrial crises (Berger et al. 2006). Tillväxtverket (2011) ranked municipalities in Sweden based on estimates of their vulnerability using dependency of individual businesses weighted by commuting; proportion of the population employed; businesses; new businesses; and an estimate of the business climate in the municipality. The highest proportion of vulnerable municipalities in Sweden (70–80 %) was located in the counties of Västmanland and Örebro, both forming the core of the Bergslagen region (Fig. 1). Around this core, Tillväxtverket (2011) showed that 21 of those genuinely vulnerable communities form a contiguous area. The gradient from the core to the periphery of Bergslagen exhibit clear trends in the average number of businesses that reached 50 % of the municipal payroll and the vulnerability rank by Tillväxtverket (2011) (Fig. 3d). Thus, the ability to manage the closure or major downsizing with increased commuting is low in the core of Bergslagen.

## Discussion

### Challenges and Opportunities for Sustainability in Bergslagen

The integrated use of ore, forests, and water in Bergslagen has had a long history of gradual development to improve effective production of iron and other metals, which formed the foundation for the national Swedish economy (Heckscher 1935–1949). This landscape history has, however, left ecological and societal footprints. Alterations of ecosystems due to pollution from mining, loss of natural forests, and alteration of streams and lakes are three key environmental issues in Bergslagen. The long history of use of natural resources thus clearly points to the need for ecosystem restoration. About 600 historic mine sites are in need of remediation in Sweden, and the total cost is estimated at two to three thousand million SEK (SEPA 2010). Some historic mine sites in Bergslagen have been remediated (Forss 1995; Karlsson and Bäckström 2003). Positive effects in terms of reduced metal fluxes and higher pH in surface waters have been obtained in the downstream area (Bäckström and Johansson 2004). At the same time, regarding the social situation, the global need for metals (Richards 2009) also provides the opportunity for new jobs associated with a revival of mining, even if at the same time high levels of municipal vulnerability is an obstacle for new inhabitants to settle down. There is also a need to adapt present management to maintain and develop cultural and natural values of forest landscapes in Bergslagen for human health and well-being. This can improve the attractiveness of municipalities to maintain their populations, and thus their economy. This requires integration both within and between ecological and social systems.

Concerning ecological systems, protection, management, and restoration of terrestrial and aquatic ecosystems need to be integrated at multiple spatial scales (Angelstam et al. 2011b; Elbakidze et al. 2011). Forest landscapes’ terrestrial ecosystems are a good example of such multiple challenges: (1) Protection of forest habitats on productive sites are underrepresented among both protected areas and in the managed landscape as a result of forest history and current forest use (Angelstam and Andersson 2001); (2) Protected areas are few and often located as isolated islands in the managed forest landscape, resulting in poor functionality of habitat networks (Angelstam et al. 2011b); (3) The proportion of forest land reserved for species conservation is still low in relation to science-based policy targets (Angelstam et al. 2011b); (4) Protection, management, and restoration measures of various kinds, and collaborations for ecological landscape planning among land owners and other stakeholders is emerging (Angelstam and Bergman 2004), but insufficient (Eriksson and Hammer 2006; Angelstam et al. 2011b). A common challenge to both terrestrial and aquatic ecosystems is to define benchmarks for ecological restoration (e.g., Degerman et al. 2004; Törnblom et al. 2011; Angelstam et al. 2013b). Additionally, effective management approaches are needed (e.g., Degerman 2008), as well as good collaboration among sectors and land owners (Angelstam et al. 2011b; Axelsson et al. 2013b).

Regarding the social system, already during the latter half of the nineteenth century major structural changes took place as mining and iron production declined (Isacson 1998a, b, 2004). From the 1970s, the restructuring of the traditional heavy industries has led to constant job losses in the traditional natural resource-dependent sectors focusing on goods. The transition from raw material production and industries to services has been and continues to be a major challenge (Isacson 1998a, b, 2004; Berger et al. 2006; Tillväxtverket 2011). Since the restructuring of industries in the 1970s to 1990s Bergslagen has lost a part of its past identity (Ågren 1998; Berger et al. 2006). The region Bergslagen is thus considered as one of the regions most struck by job losses in Sweden, together with the sparsely populated inner part of Norrland in the north (Jakobsson 2009; Tillväxtverket 2011). Currently, however, Bergslagen is in the process of finding new ways for development (Isacson 1998a, b, 2004; Berger et al. 2006; Andersson et al. 2012a), including ecological and cultural heritage values as a base for rural development (Jakobsson 2009). Bergslagen is hence used as a brand (Heldt Cassel 2008; Jakobsson 2009), even if today’s administrative borders subdivide the historic Bergslagen region. In response to this several organizations and civic initiatives aim at sustainable development and sustainability in the informal Bergslagen region (Elbakidze et al. 2010; Andersson et al. 2012a; Axelsson et al. 2013b).

### Need for Novel Governance Arrangements

The identity of Bergslagen was formed through a long history of natural resource use in terms of ore, forest, and water as a base for industrial development. Industries historically provided jobs and took care of many societal functions (Isacson et al. 2009). People were employed as workers and there was neither much need nor space for individual entrepreneurship (Ågren 1998). This is linked to limited social and cultural capital, which has shaped people and communities, and there is even a Swedish word that captures the mental status of local communities with a focus on mining and metallurgy (“bruksanda”) (Ekman 1996; Bergdahl et al. 1997).

Economic globalization, energy production (biomass, water and wind), and climate change are current issues that affect landscape management and governance in Bergslagen. Additionally, with increasing mineral prices internationally there is a renewed interest to resume mining operations in Bergslagen. This requires collaboration among actors from the prospecting phase to the establishment of a new mine to its closing, including minimizing the environmental impact, and restoring the mining site after ore extraction. However, the realization of sustainable mining includes not only mines themselves, but also a need to support societal infrastructures in general (Richards 2009). To deal with this complex governance issue Young (2013) argued for applied institutional analyses as institutional diagnostics. This has much in common with other applied sciences, and aims at bridging the disconnect between the worlds of analyses and praxis by joint “identification of key features of specific problems and the application of relevant propositions to guide the process of crafting governance systems to solve these problems case by case” (Young 2013).

Development is generally considered to be supported by improved social capital and an associated level of entrepreneurship (Knack and Keefer 1997; Bruckmeier and Tovey 2009). Besides the large environmental debt due to intensive use of land and water, among the problems facing Bergslagen are a limited entrepreneur tradition and a low level and standard of education. Many young people have moved out from the region because of the lack of jobs (Jakobsson 2009). Also, cuts in the state budget, deregulation, and privatization have often hit rural areas harder than urban centers (SOU 1997). Strengthening regional identity is an important part of regional development. Cultural and remaining ecological values of forest landscapes in Bergslagen are attractive to people. This has led to a wave of seasonal and weekly amenity migration of people seeking recreation and better quality of life. Some move permanently from densely populated regions in Europe, while others split their time between recreation in Bergslagen and working remotely in Stockholm and other cities in central Sweden. Thus, cultural heritage has been promoted to strengthen regional identity in Bergslagen (Jakobsson 2009). However, efforts are more concentrated on commercial use and packaging of natural and cultural heritage as a so-called experience industry in order to create an attractive image of Bergslagen for tourists (Jakobsson 2009). Additionally, learning processes that identify able entrepreneurs and real development opportunities need to be encouraged. The use of industry buildings for new purposes is a good example of how old infrastructures can be used in new ways (Geijerstam 2007). We argue that these efforts toward using cultural heritage as an infrastructure for local and regional development based on tourism and amenity migration should, however, be matched by studies of residents’ and visitors’ preferences. The location of the Bergslagen region immediately adjacent to south-central Sweden’s urban centers offers good opportunities for reviving the historical interdependency of rural and urban regions (Eimermann et al. 2012).

### Learning from Comparative Studies

A current challenge in Bergslagen is to encourage development of adaptive management and governance in social–ecological systems at relevant scales. Concerning ecosystem restoration this requires development of evidence-based knowledge about the composition, structure, and function of near-natural ecosystems as benchmarks for assessment of sustainability (see Angelstam et al. 2013b), and collaboration models that allow different landscape stakeholders to work together at multiple levels (Axelsson et al. 2013b). Transparent information about different dimensions of sustainability is therefore necessary (Andersson et al. 2012a, b). How have places with other histories coped with this?

The combination in the same region of minerals in the bedrock, water providing kinetic energy, and wood for fuel and construction in the Swedish Bergslagen region made it the main global producer of iron during the seventeenth century (Hildebrand 1992). The same combination of resources triggered a 1000-year history in the Harz Mountains in Germany, and in the mid-Ural Mountains in Russia from the early eighteenth century (Portal 1950; Hildebrand 1992). These three regions represent places located in different historical phases of natural resource use (Mavor 1925), and have different trajectories of developing governance arrangements. Like Bergslagen, both Harz and Ural are currently faced with increased demands of delivering natural resources and to produce value-added products, as well as to sustain ecological, social, and cultural values.

The Upper Harz was once one of the most important mining regions in Germany (Fleisch 1983; Ließmann 1997; Wagner and Wellmer 2009). The last mine in the Upper Harz—the Wolkenhügel Pit in Bad Lauterberg—was closed in 2007. For centuries the main products were firewood, timber, and charcoal. Forest management was established in 1712. Until about year 1800 practically only beech *Fagus sylvatica* and oak *Quercus robur* were used, and coppice forestry dominated. From the 1790s spruce began to be planted. The commercially managed areas are mainly monocultures of Norway spruce, a legacy from the mining history with its high demand for wood. Today, heavy metal residues, biodiversity conservation, and rural development are three key challenges (Hauhs and Lange 2000). Management of the forest landscape in the Harz is currently focused on how to deal with heavy metal depositions from nearly 500 years of intensive mining operations, returning some of the planted spruce (which represents almost 90 %) to deciduous forests, red deer damaging the forest, reforesting after wind damage, and bark beetle infestation. As a consequence of both ecological problems (Jansen et al. 2002; Hauhs and Lange 2010a, b) and changing values in society (Lehman 2001) the forest administration in Lower Saxony took an initiative to transform forestry practices. Protection, conservation, and recreation are coordinated today in an ecological planning process called Löwe (Langfristische Ökologisches Walderneuering) in the Harz. This is applied to 54 000 ha of state forests, is recommended to municipal forests covering more than 3000 ha, and includes a 17 000 ha national park with low forest management intensity. Harz is a major tourist destination, including geotourism (Torabi Farsani et al. 2011), which involves conflicts between traditional industrial and new service-related products (Job 1996).

The emergence of industrial metal production in the Urals in the early eighteenth century was linked to the huge deposits of shallow high-quality iron ore and vast expanses of virgin forests providing wood (Lepechin 1802; Attman 1981; Ågren 1998; Evans and Rydén 2007; Alekseev and Gavrilov 2008). The construction of iron-based industry in the Urals in the eighteenth and the first half of the nineteenth centuries involved the establishment of about 200 iron-producing plants, dams on the rivers, intensive deforestation to produce charcoal, and clearing of forests for arable land and meadows. Forest management began in the eighteenth century by dividing forests into management units, harvesting plots with blocks left for natural regeneration, and preventing forest fires (Shishonko 1887). The arrival of the industrial revolution in the late eighteenth and nineteenth centuries with steam engines and machine industry led to a sharp increase in metal production, and increased the pressure on ecosystems (Gavrilov 2005). After the First World War and the civil war, which were linked to economic collapse and paralysis of transport and fuel limitation, the role of wood in the country’s fuel balance increased dramatically (Alekseev and Gavrilov 2008). During Second World War, in 1941–1942, Russia’s largest companies were evacuated from the westernmost areas of Russia to the Urals, which further complicated the environmental situation in the region. This development has impacted social–ecological systems. The forest area has gradually decreased in the Urals (Boreyko 1990), and tree species composition has changed. Intact forest landscapes with naturally dynamic forests are found only in the northern periphery of the Ural region. In the late nineteenth and early twentieth centuries metal production in the Urals had dramatically increased pollution and wastes from industrial production (Gavrilov 1997). Especially harmful were sulfur-containing gases (Gavrilov 1992). In response to environmental issues, non-governmental organziations formed a social movement in the Urals that included forest protection and nature conservation in their agenda already at the turn of the nineteenth and twentieth centuries. By contrast, the modern environmental movement in the Urals is primarily a professional movement that challenges specific and narrow objectives.

Studies of countries and regions with different histories (Lehtinen et al. 2004; Angelstam et al. 2013a) is a useful approach to understand how natural resource use and its consequences develop, which institutions govern this, and what the consequence are. Comparisons of regions and countries with a shorter and longer history of use of natural resources than in Bergslagen, and with different governance arrangements, such as in Russia and Germany, respectively, can thus both validate results from single case studies, and demonstrate the role of different contexts (Angelstam et al. 2011a, 2013c).

## Conclusions

Comprehensive knowledge about the history of landscapes as social–ecological systems clearly contributes to understanding the current state of different aspects of sustainability. This knowledge production requires collaboration among scholars from different disciplines, and practitioners. Additionally, to generate knowledge about history useful in contemporary governance and management towards sustainable landscapes and regions requires improved spatial planning to cope with conservation, management, and restoration of consequences of mining, forestry and use of water, as well as rural development. As illustrated by the brief comparison of multiple social–ecological systems, comparative studies can provide relevant knowledge and insights for this.

## Electronic supplementary material

Below is the link to the electronic supplementary material.

Supplementary material 1 (PDF 30 kb)

## Footnotes

1 See http://www.raa.se/cms/fornsok/start.html.

2 See http://sn.vic-metria.nu/skyddadnatur/index.jsf.

3 See http://www.smhi.se/klimatdata/hydrologi/ladda-ner-hydrologiska-data-1.20127.
