# Supplementary material for: Learning About the History of Landscape Use for the Future: Consequences for Ecological and Social Systems in Swedish Bergslagen
Source: Ambio. 2013 Mar 10;42(2):146–59. doi: 10.1007/s13280-012-0369-z (PMC3593034; doi:10.1007/s13280-012-0369-z)
Supplement: Supplementary file 1 — Supplementary material 1 (PDF 30 kb) [file 13280_2012_369_MOESM1_ESM.pdf]

Electronic Supplementary Material

Learning about the history of landscape use for the future: consequences for ecological and social systems in Swedish Bergslagen

Per Angelstam, Kjell Andersson, Maths Isacson, Dmitri V. Gavrilov, Robert Axelsson, Mattias Bäckström, Erik Degerman, Marine Elbakidze, Elena Yu. Kazakova-Apkarimova, Lotta Sartz, Stefan Sädbom, Johan Törnblom

Table S1 Different definitions of Bergslagen used to compile the core of Bergslagen with parishes as minimum mapping units

Id	Description	Reference	Type of data
1	Bergslager in Sweden	Atlas of Sweden's Bergslager Geijerstam and Nisser (2011)	Printed map
2	Bergslagen border	Nordisk familjebok (1925)	Printed map
3	Mining areas	Seebass (1928)	Printed map
4	Bergslagen Geological extent 1916	Seebass (1928)	Printed map
5	Iron industry 1919	Seebass (1928)	Printed map
6	Culture Geographical province 1918	Seebass (1928)	Printed map
7	Rural area Bergslagen 1918	Seebass (1928)	Printed map
8	Rural area Bergslagen 1921	Seebass (1928)	Printed map
9	Region Bergslagen 1925	Seebass (1928)	Printed map
10	Topographic Bergslagen 1927	Seebass (1928)	Printed map
11	Agricultural census Bergslagen	Jordbruksräkningen (1932)	Description
12	Bergslagen in sense of legal framework for the iron works at the time, "brukslagstiftning"	Heckscher (1935–49)	Printed map
13	Bergslagsgruppen	Arpi (1951)	Printed map
14	Bergslagsområdet	Carlquist and Carlsson (1962)	Printed map
15	Bergslagen as agricultural region	W:son Ahlmann et al. (1966:352)	Printed map
16	Sweden's mines and mineralizations	SGU (2011)	Printed map
17	Bergslaget – member municipalities	www.bergslaget.com	Description
18	Parishes containing places with businesses having Bergslagen in their name	Eniro, www.eniro.se, retrieved 23 April 2012	Analysis
19	Trains in Bergslagen - Parishes within a buffer of 20 km from railways.	Using GIS we made a 20-km buffer around the railway line (Linjekarta, www.tagibergslagen.se, retrieved 23 April 2012)	Analysis
20	Bergskraft Bergslagen.	Based on the map National bedrock data, SGU 2007.	Digital map
21	Ekomuseum Bergslagen	www.ekomuseum.se	Digital map
22	Leader Bergslagen	www.leaderbergslagen.se	Digital map

References

Carlquist, G., and J. Carlsson. 1962. Väderleksförutsägelser. [Weather forecasts]. *Svensk Uppslagsbok* 31: 873 (in Swedish).

Geijerstam, J., and M. Nisser. 2011. *Swedish Mining and Metalworking – Past and Present. National Atlas of Sweden.* Stockholm: Norstedts Förlagsgrupp AB.

Jordbruksräkningen. 1932. Stockholm: Statistiska centralbyrån (in Swedish).

Heckscher, E. 1935–49. *Sveriges ekonomiska historia från Gustav Vasa. [Sweden's economic history from Gustav Vasa].* Stockholm: Albert Bonniers förlag (in Swedish).

Nordisk Familjebok. 1925. *Bergslagen*. Malmö: Nordisk Familjeboks förlag (in Swedish).

Seebass, F. 1928. *Bergslagen: Versuch einer kulturgeographischen Beschreibung und Umgrenzung. [Bergslagen: An attempt to human geogegraphical description and delimitation]*. Greifswald: J. Abel, g.m.b.h. (in German).

W:son Ahlmann, H., G. Arpi, G. Hoppe, and C. M:son Mannerfelt. 1966. *Sverige. Land och folk [Sweden: Land and people].* Stockholm: Natur och Kultur (in Swedish).

SGU. 2007. National Bedrock Data, Geological Survey of Sweden, 13p, Uppsala (in Swedish).

SGU. 2011. Statistics of the Swedish Mining Industry 2010. Periodiska publikationer 2011:2. Uppsala: Geological Survey of Sweden.
